# Attitudes toward climate change risk among older people: new evidence from the English Longitudinal Study of Ageing

**DOI:** 10.1093/geronb/gbag029

**Published:** 2026-03-10

**Authors:** Giorgio Di Gessa, Paola Zaninotto

**Affiliations:** Department of Epidemiology and Public Health, University College London, London, United Kingdom; Department of Epidemiology and Public Health, University College London, London, United Kingdom; (Social Sciences Section)

**Keywords:** Climate risk, Climate perception, Climate beliefs, England, Profiles

## Abstract

**Objectives:**

This study investigates the diversity of attitudes toward climate change risk (ACCR) among older adults in England. This demographic, both vulnerable to climate impacts and influential in shaping climate policy, has often been overlooked in terms of its specific ACCR. The study aims to identify distinct attitudinal profiles and explore the sociodemographic, economic, health, and civic factors associated with them.

**Methods:**

Using data from Wave 11 (2023–2024) of the nationally representative English Longitudinal Study of Ageing, we analyzed responses from 6,572 individuals aged 50 and older. Latent class analysis was employed to identify typologies of ACCR based on 6 climate-related statements. Multinomial logistic regression was used to examine associations between class membership and individual characteristics.

**Results:**

Five distinct ACCR profiles were identified: “Highly engaged with climate change risk” (30.3%), “Engaged with CCR” (31.3%), “Risk-aware but fatalistic” (11.1%), “Ambivalent/uncertain about CCR” (21.5%), and “CCR dismissive” (5.8%). Younger age, higher education, greater financial resources, and higher levels of public engagement were associated with a higher likelihood of being “Highly engaged” about climate change. Conversely, lower education, economic hardship, and lack of civic engagement were linked to “ambivalent/uncertain” attitudes. Notably, older adults were more likely to be risk-aware but fatalistic.

**Discussion:**

Contrary to common assumptions, most older adults are engaged with CCR, but there is notable heterogeneity, with ∼27% reporting ambivalent/uncertain, or dismissive views. Inclusive and effective climate policy should recognize this diversity, employing outreach and communication strategies that stress personal relevance and actionable solutions, especially targeting those with ambivalent/uncertain views.

Climate change is widely recognized as the greatest threat to societies and global health in the 21st century, with 3.6 billion people in the world who live in areas highly susceptible to climate change and a conservative estimate of 250,000 additional yearly deaths by the 2030s due to climate change impacts (World Health Organization, 2024). Climate change is primarily caused by increased greenhouse gas levels in the atmosphere, mainly due to human activities (e.g., the burning of fossil fuels, deforestation, and increased livestock farming) that alter the environment and have direct repercussions on our lives. Suitable, effective, and well-funded policies are required to minimize the adverse health impacts of climate change (Romanello et al., 2024). Other key factors in achieving emissions levels compatible with remaining well below the maximum of 2 °C of warming by the end of the 21st century include widespread public support and behavioral change—that is, how seriously, urgently, and actionably individuals perceive the risks of climate change. As numerous studies have shown, low perceptions of climate change risk can hinder both public support for climate policies and personal engagement in mitigation and adaptation efforts (Spence et al., 2011; Weber, 2010), with previous research suggesting a link between public opinion and climate policy (Tjernström & Tietenberg, 2008; Vandeweerdt et al., 2016). However, research on the nuances and heterogeneity of attitudes to climate change risk (ACCR), including perceived likelihood and severity of climate impacts, worries and concerns, and value judgments about the dangers posed by climate change and the need for action (Van der Linden, 2017), remains limited, particularly among older people.

Older adults represent a group whose relevance to the climate crisis is twofold: they are disproportionately vulnerable to its effects, and they possess significant potential to influence climate outcomes. They are often more severely affected by climate-related events such as storms, wildfires, flooding, and heat waves (Filiberto et al., 2009; Prina et al., 2024). Physiologically, older adults face heightened risks from heatwaves, air pollution, and climate-exacerbated disease, owing to age-related health conditions and reduced adaptive capacity (Kazi et al., 2024; Paavola, 2017). Extreme weather events often lead to excess mortality, with the majority of deaths occurring among older populations (Ballester et al., 2023; Bunker et al., 2016). Even floods are associated with increased mortality among older individuals and in communities with larger proportions of older residents (Lynch et al., 2025; Yang et al., 2023), often due to a combination of reduced mobility, greater risk of drowning, and disruptions to essential healthcare services. Climate change and climate-related events may also affect older people’s social and mental well-being (Lawrance et al., 2022; Song et al., 2025). It is often hypothesized that older people might be at higher risk of social isolation, reduced support networks, and challenges accessing information, healthcare services, or assistance during extreme weather events, although they can also show greater emotional resilience following disasters (Strough et al., 2024).

Yet, older people should not only be considered passive victims of the environmental threats posed by climate change but also recognized as key players who can contribute to climate adaptation and advocacy. Older people are often accused of contributing to greenhouse gas emissions (Han & Ahn, 2020), although research indicates mixed evidence on their contribution to carbon emissions, given the heterogeneity of older people and their lifestyles (Fan et al., 2021; Pais-Magalhães et al., 2022), with per-capita CO_2_ emissions that tend to decline after the age of 60 (Zagheni, 2011). Moreover, many studies suggest that climate change and the environment are top issues across all generations, with age-group differences often dependent on whether questions refer to climate change beliefs, perceptions, or emotions (Milfont et al., 2021; Poortinga et al., 2023). For example, Poortinga et al. (2023) found that younger generations in the UK felt the negative emotions of fear, guilt, and outrage more strongly than older generations, whereas the latter were more inclined to believe that they were already experiencing the effects of climate change. A UK 2024 poll showed that about 57% of adults reported climate change and the environment as an “important issue facing the UK,” with no significant differences across age groups (Office of National Statistics, 2024). However, engagement in volunteering activities related to the environment, including donating money, contacting an elected official, volunteering, or attending a rally, remains lower among older people (Tyson et al., 2021), showing the underutilized potential that older people can play in climate change action (Pillemer et al., 2021). This is particularly important given that older people are a growing demographic with significant voting power and community influence. In the EU, for example, the number of older people (defined here as those aged 65 years or more) is expected to rise from 94 million in 2024 to 130 million by 2050, with the share of older people expected to grow from 21% to 29% in the same period (Eurostat, 2025). For policies that seek to protect the environment (e.g., in favor of climate change mitigation measures), it is therefore essential to have the support and engagement of older people who, in many liberal democracies, show a high level of political participation, including a higher propensity to vote and higher turnouts than younger voters (Ahlfeldt et al., 2022; Goerres, 2007).

Despite the links between aging and climate change, relatively few studies have focused on older people’s attitudes toward climate change risk (Ni et al., 2025), often because only a limited number of existing aging studies included questionnaires specifically addressing climate change and attitudes toward this issue (Wu et al., 2025). A meta-analysis of studies and data sets that include questions about beliefs about climate change suggests that age had a relatively small negative effect, with younger people reporting stronger beliefs in climate change (Hornsey et al., 2016). Using data from a 2018 online survey conducted among British citizens aged 18 and over (*N* = 787), and questions on three dimensions of climate change (belief in and concern about climate change, issue salience, and support for government action) that were summarized using latent class analysis (LCA), Crawley et al. (2020) found that younger people were more likely to be highly or moderately engaged with this issue, whereas older people were more likely to be climate change deniers. Similarly, using LCA on nine measures of environmental attitudes collected among British adults, Rhead et al. (2018) found that the “disengaged” class had the highest proportion of people aged 60 and older. Recently, a UK study conducted among household members aged 16 or above that used cluster analysis to group respondents based on their answers to five questions regarding attitudes and beliefs toward the seriousness, urgency, and preventability of climate change, found age to be a weak and inconsistent predictor of membership in different profiles of ACCR (Liu et al., 2022). However, most of these studies assume that attitudinal profiles are constant across the entire population, failing to acknowledge that there may be significant variations in ACCR among different age groups. Similarly, using broad age categories (e.g., those aged 55 and older) is problematic because it neglects the heterogeneity among older adults, including differences in life stages, opportunities, experiences, and health and financial needs. More research into how older adults perceive climate risks, how their attitudes cluster, and what factors influence these patterns is particularly important at a time when older people’s perceptions of and emotional engagement with climate change should be integrated into the development and dissemination of climate action to ensure the highest participation in climate change adaptation and mitigation efforts.

This study, therefore, aims to provide an overview of the typologies of attitudes toward climate change risk, specifically among older adults, moving beyond simplistic generational comparisons to understand the extent of heterogeneity among this demographic group. As is increasingly done in attitudinal studies on climate change risk (Crawley et al., 2020; Kácha et al., 2022; Liu et al., 2022; Milfont et al., 2024; Rhead et al., 2018), we will consider multiple dimensions of climate change simultaneously to capture cognitive (beliefs about climate change and its causes), affective (the degree of worry or concern), and evaluative (perceived salience and severity) dimensions. Combining several questions simultaneously enables researchers to gain a deeper understanding of knowledge about climate change, emotional engagement with the issue, and both personal and societal implications. This, combined with the use of LCA (a person-centered approach that identifies qualitatively distinct groups that share similar patterns across belief, concern, and salience), also allows a better segmentation of people into distinct attitudinal profiles, which, in previous works carried out in the general population, have ranged from “skeptical” about the issue to “extremely worried” groups (Crawley et al., 2020; Kácha et al., 2022; Liu et al., 2022; Milfont et al., 2024; Rhead et al., 2018). Once these groups of differential attitudes toward climate change risk are identified among older people, we investigate the factors associated with the likelihood of belonging to such groups. In particular, we focus on sociodemographic and economic characteristics, as well as social and political engagement, as these factors have often been reported to be associated with environmental concern groups. In this study, we do not hold any predetermined hypotheses about the role of these factors, given that we do not know a priori how many groups, or which ones, will be identified among older English people. However, in line with previous findings, we expect respondents with higher socioeconomic backgrounds and greater engagement to exhibit greater awareness of and responsiveness to climate change risks.

## Method

### Study population

Data are from the English Longitudinal Study of Ageing (ELSA), an ongoing multidisciplinary longitudinal survey of individuals aged 50 and older. The study began in 2002/2003, when around 12,000 respondents were recruited to provide a representative sample of the population aged 50 and over living in private households in England (household response rate was 70%). ELSA has periodically refreshed its sample with new participants to ensure the study remains representative of individuals aged 50 and older in England (Steptoe et al., 2013). Since its inception, ELSA has collected information biennially, with the sequence interrupted by the COVID-19 pandemic; the latest available data (Wave 11) was collected between October 2023 and October 2024. Further details of the survey’s sampling frame and methodology can be found at www.elsa-project.ac.uk. All waves of ELSA received ethical approval, with the latest wave approved by the South Central—Berkshire Research Ethics Committee (23/SC/0112). Informed consent was obtained from all participants. All data are available through the UK Data Service (SN 5050). For this study, we focused on respondents aged 50 and older who were interviewed in Wave 11 (*N *= 7,747), as this was the first wave of ELSA that introduced a module on attitudes and beliefs toward the seriousness, urgency, and preventability of climate change. The analytical sample consisted of respondents who returned the self-completion questionnaire, which included questions about attitudes toward climate change risk (*N *= 6,738; 87% of the sample), with available information on all variables of interest (*N *= 6,572; 85%).

### Main measurements of interest

#### Attitudes to climate change risk

Respondents were asked to indicate their agreement with six statements regarding opinions on climate change using a 5-point Likert scale (1 = Strongly Agree to 5 = Strongly Disagree). Although we acknowledge that all six statements contain elements that simultaneously capture beliefs, worry, and seriousness, they best serve as items of cognitive (“People in the UK will be affected by climate change in the next 30 years” and “The so-called ‘environmental crisis’ facing humanity has been greatly exaggerated”), affective (“Climate Change is beyond control, it’s too late to do anything about it” and “The effects of Climate Change are too far in the future to really worry me”), and evaluative (“If things continue on their current course, we will soon experience a major environmental disaster” and “I am prepared to pay more for environmentally friendly products”) dimensions.

#### Covariates

In line with previous studies, we accounted for a wide range of respondents’ characteristics, including sociodemographic, economic, health-related, and social/civic engagement factors. Sociodemographic variables included age groups (50–59, 60–69, 70–79, or 80 and older), sex (male vs. female), marital status (married or cohabiting vs. unmarried), and children (none vs. one or more living children). As socioeconomic factors, we considered education and a self-assessment of financial well-being. Educational level was recoded into three categories (low, middle, high) using the International Standard Classification of Education (http://www.uis.unesco.org/), where low education refers to below secondary education, and a high educational level is defined as having a university education or above. Respondents were also asked how they (and their partner) were getting along financially these days, with six options ranging from “manage very well” to “have severe financial difficulties.” In our study, we distinguish between those who manage “very well,” “quite well,” and those who answered anything between “getting by alright” and “having severe financial difficulties.” Health variables included self-perceived health and depressive symptomatology. Self-rated health (SRH) was measured on a 5-point ordinal scale (excellent, very good, good, fair, or poor). The five SRH items were dichotomized into “fair or poor” versus better health. For mental health, ELSA included an abbreviated eight-item version of the Center for Epidemiologic Studies-Depression scale (CES-D) (Radloff, 1977). Respondents are asked whether they had experienced any depressive symptoms, such as restless sleep or being unhappy, in the week before the interview, with those reporting four or more classified as having elevated depressive symptoms (Turvey et al., 1999). As an indicator of aspects of personality, we considered optimism, measured by the sum of the ratings of two statements (“I feel that life is full of opportunities” and “I feel that the future looks good for me”), scored on a 4-point scale (from 3 = “Often” to 0 = “Never”) (Chilcot & Hackett, 2024), with those scoring four or above classified as overall optimistic. Social/civic engagement was captured through several dichotomous variables, including whether the respondent had voted in the latest general election, whether they were a member of a political party, trade union, or environmental group, whether they were a member of a religious group, and whether they had volunteered in the previous month. As indicators of awareness and concern about climate change, as well as willingness to positively contribute to the environment (Pagliuca et al., 2022), we included a variable indicating whether respondents had environmentally friendly household items (such as solar panels, heat pumps, wind turbines, and electric cars). Although environmentally friendly household items may reflect behavioral manifestations of environmental concern, we treat them as correlates rather than components of ACCR. Whereas ACCR questions focus explicitly on beliefs and perceived risks, household items reflect tangible behavioral adoption and their possession could, in turn, be influenced by affordability, policy incentives, and infrastructure (rather than merely by one’s levels of beliefs and worries). Finally, as previous studies have shown an association between higher temperatures and climate change belief and concern (Sugerman et al., 2021), we also controlled for seasonality and whether the interview took place in the summer or not.

### Statistical analysis

We conducted two steps of analysis. First, we employed LCA to group individuals based on their patterns of responses to indicators of ACCR. LCA identifies homogenous subgroups (classes), which fit the data well and account for associations among the observed variables. We opted for LCA because the purpose of this study was to identify qualitatively distinct attitudinal profiles rather than underlying continuous dimensions. Moreover, we retained the full Likert scales for each ACCR item to maintain meaningful gradations that are important for differentiating levels of beliefs, worry, and the seriousness of climate change, including neutral stances. For each subject, the LCA model provides the probability of belonging to each identified group and assigns the subject to the subgroup with the highest probability. To determine the number of groups within our sample, we tested models with up to six classes. We compared them using absolute and relative model fit statistics, including the Pearson chi-square statistic, the bootstrap likelihood ratio test, and the Bayesian information criterion (BIC). For each of these, lower scores indicate (relatively) better fitting models. Moreover, we considered the entropy index to assess classification quality, with values approaching 1.0 indicating a favorable classification (Celeux & Soromenho, 1996), the average latent class probability for each group (>80%); group size (and the avoidance of groups smaller than 5% that may lead to lack of reproducibility of the results); and the interpretability of the distinctive groups (Weller et al., 2020). For each latent class model, we specified 1,000 random sets of starting values, 500 optimizations, and 100 iterations in the final stage to ensure replication of the best log-likelihood and that solutions are not derived from local maxima.

Second, we used a multinomial regression model to identify sociodemographic, economic, health, and social/civic engagement factors associated with different classes of ACCR. To facilitate the interpretation of the results, we present average marginal effects (AMEs), which, due to the categorical nature of our outcomes and covariates, should be interpreted as the average change in the probability of the outcome across the categories of the covariates being examined. AMEs are summary measures that estimate a predictor variable’s partial effect (in percentage points, pp) on the outcome. Latent summaries and LCA were estimated using Mplus 7.3; data management and statistical analyses were performed using Stata/MP 18.5.

## Results

### Descriptive statistics

Table 1 describes the characteristics of the study sample in 2023/2024. Just over half of the participants were female (56.3%), and approximately one-third were aged 60–69. Most participants were partnered (64.5%) and had children (83.9%). Regarding socioeconomic characteristics, 30.5% of respondents had high levels of education, with 46% reporting getting along very well financially. Most ELSA respondents reported at least good SRH (72.7%) and no depressive symptomatology (86.1%). The vast majority of participants voted in the latest general elections (83.1%), with 12.6% being members of a political party, trade union, or environmental group, 15.8% members of religious groups, and 16.8% engaging in volunteering. One in seven respondents has environmentally friendly household items.

**Table 1:** Descriptive statistics of ELSA respondents (2023/2024).

Characteristics	% (*N*)
**Age**	
50–59	21.8 (1,431)
60–69	32.9 (2,163)
70–79	31.6 (2,074)
80 and older	13.7 (904)
**Female**	56.3 (3,704)
**Partnered**	64.5 (4,242)
**No children**	17.0 (1,117)
**Education**	
Low	20.3 (1,335)
Middle	49.1 (3,228)
High	30.6 (2,009)
**Financially, gets along**	
Very well	46.2 (3,036)
Quite well	30.7 (2,018)
Alright or with difficulties	23.1 (1,518)
**≥Good self-rated health**	72.9 (4,794)
**≤3CES-D depressive symptoms**	86.1 (5,664)
**Optimistic outlook**	71.8 (4,720)
**Member of a political party, a trade Union, or an environmental group**	12.8 (840)
**Voted in the last general election**	83.1 (5,464)
**Member of a church or other religious group**	15.8 (1,040)
**Voluntary work in the previous month**	17.0 (1,117)
**Has environmentally friendly household items**	14.8 (972)
**Interviewed in Summer**	28.8 (1,890)

*Note*. *N *= 6,572. ELSA, W11 (2023–2024).

Table 2 shows ACCR among all ELSA respondents. Overall, only 6.2% of respondents strongly agree that climate change is beyond control and that it is too late to do anything about it, with an even smaller percentage of older people (4.6%) strongly agreeing that the effects of climate change are too far away in the future to worry them or that the environmental crisis has been exaggerated. Almost half of the respondents (47.4%) strongly agree that people in the UK will be affected by climate change in the next 30 years, with a quarter strongly agreeing that, without action, the UK will experience a major environmental disaster. About 15% of older people disagree or strongly disagree that they are ready to pay more for environmentally friendly products.

**Table 2:** Descriptive statistics of attitudes to climate change risk, ELSA (2023/2024).

Statement	Strongly agree	Tend to agree	Neither agree nor disagree	Tend to disagree	Strongly disagree
**People in the UK will be affected by climate change in the next 30 years**	47.4%	34.8%	11.6%	2.9%	3.3%
**The so-called “environmental crisis” facing humanity has been greatly exaggerated**	4.5%	10.8%	24.3%	27.0%	33.4%
**Climate change is beyond control, it’s too late to do anything about it**	6.1%	24.3%	25.0%	28.7%	15.9%
**The effects of climate change are too far in the future to really worry me**	4.5%	12.9%	18.9%	28.6%	35.1%
**If things continue on their current course, we will soon experience a major environmental disaster**	25.8%	38.4%	25.4%	7.1%	3.3%
**I am prepared to pay more for environmentally friendly products**	18.4%	39.7%	27.1%	9.2%	5.6%

*Note*. The table shows the extent to which respondents agree or disagree with each statement on climate change risks. *N* = 6,572. ELSA, W11 (2023–2024).

### Classification of attitudes to climate change risk

We classified respondents’ attitudes toward climate change into five homogenous subgroups, as these provided the best model fit (as defined above, see Supplementary Table 1). Table 3 presents the conditional probabilities of responding to each indicator variable within each class (i.e., item response probabilities), the percentage of the sample in each latent class, and interpretative descriptive labels summarizing the complex patterns of response observed within each class. For instance, 30.3% of respondents scored high on cognitive, affective, and evaluative dimensions of climate change risk (hereafter, “Highly engaged with climate change risk”). These respondents show the highest levels of strong agreement or disagreement with most statements, understanding the issue, seeing it as severe and important enough to act on, and being prepared, for instance, to pay more for environmentally friendly products. The second group is also informed about climate change and worried about related risks, seeing this as an urgent and serious issue. Still, respondents in this group are less vigorous in their level of agreement or disagreement and are, therefore, labeled as “Engaged with climate change risk” (31.3%). For example, only 33% strongly agreed that people in the UK will be affected by climate change in the next 30 years (and 59.5% tended to agree) compared to 94% in the “highly engaged with climate change risk” group. Class 3 is composed of respondents who are “Risk-aware but fatalistic” (11.1%); these respondents score high on cognitive and evaluative dimensions of climate change risk, but endorse statements that it is too late to act and too distant in the future to worry about. About one in five respondents (21.5%) is labeled as “Ambivalent/uncertain about climate change risk”; these respondents show an ambivalent/uncertain climate risk profile, characterized by consistent midpoint (“neither agree nor disagree”) responses across cognitive, affective, and evaluative items. Finally, 5.8% of older people were classified as “Climate change risk dismissive”: these respondents tend to strongly disagree that climate change will affect the UK in the next 30 years (40.7%) or lead to a major environmental disaster (52.8%), endorsing the view that the environmental crisis has been greatly exaggerated (41.7%) and showing low willingness (with 39.4% strongly disagreeing) to pay more for environmentally friendly products.

**Table 3:** Conditional response probabilities for each statement about climate change risks in the five latent classes.

Statement	Level of dis/agreement	Class 5	Class 1	Class 4	Class 2	Class 3
		Highly engaged with climate change risk (30.3%)	Engaged with climate change risk (31.3%)	Risk-Aware but fatalistic (11.1%)	Ambivalent/uncertain about climate change risk (21.5%)	Climate change risk dismissive (5.8%)
**People in the UK will be affected by climate change in the next 30 years**	Strongly agree	0.942	0.328	0.665	0.034	0.071
	Tend to agree	0.038	0.595	0.317	0.502	0.118
	Neither agree nor disagree	0.005	0.03	0.011	0.432	0.208
	Tend to disagree	0.002	0.031	0.007	0.031	0.196
	Strongly disagree	0.014	0.016	0.001	0.001	0.407
**The so-called “environmental crisis” facing humanity has been greatly exaggerated**	Strongly agree	0.013	0.001	0.102	0.028	0.417
	Tend to agree	0.002	0.063	0.234	0.236	0.195
	Neither agree nor disagree	0.008	0.224	0.216	0.666	0.065
	Tend to disagree	0.097	0.607	0.266	0.07	0.100
	Strongly disagree	0.879	0.105	0.182	0.000	0.222
**Climate change is beyond control, it’s too late to do anything about it**	Strongly agree	0.063	0.004	0.275	0.019	0.107
	Tend to agree	0.226	0.201	0.553	0.21	0.088
	Neither agree nor disagree	0.086	0.206	0.152	0.619	0.175
	Tend to disagree	0.278	0.502	0.012	0.137	0.241
	Strongly disagree	0.347	0.087	0.008	0.015	0.389
**The effects of climate change are too far in the future to really worry me**	Strongly agree	0.024	0.006	0.208	0.018	0.168
	Tend to agree	0.008	0.079	0.417	0.233	0.118
	Neither agree nor disagree	0.007	0.098	0.168	0.580	0.199
	Tend to disagree	0.132	0.586	0.17	0.159	0.157
	Strongly disagree	0.828	0.231	0.035	0.010	0.359
**If things continue on their current course, we will soon experience a major environmental disaster**	Strongly agree	0.623	0.046	0.469	0.005	0.03
	Tend to agree	0.335	0.621	0.438	0.186	0.005
	Neither agree nor disagree	0.034	0.267	0.086	0.661	0.151
	Tend to disagree	0.005	0.064	0.007	0.145	0.285
	Strongly disagree	0.003	0.002	0.000	0.004	0.528
**I am prepared to pay more for environmentally friendly products**	Strongly agree	0.466	0.046	0.156	0.02	0.113
	Tend to agree	0.400	0.549	0.410	0.22	0.177
	Neither agree nor disagree	0.092	0.305	0.299	0.496	0.157
	Tend to disagree	0.022	0.078	0.069	0.201	0.158
	Strongly disagree	0.021	0.023	0.066	0.064	0.394

*Note*. Entropy statistics for this classification and class assignment probabilities for each class are available in Supplementary Table 1. ELSA, Wave 11 (2023/2024).

### Factors associated with classes of attitudes to climate change risk

Table 4 presents the results, expressed in terms of AMEs, from the multinomial logistic regression analysis of demographic, socioeconomic, health, and civic and social participation factors associated with different classes of attitudes toward climate change risk. The results suggest age-group differences, particularly between the “Highly engaged with climate change risk” and the “Ambivalent/uncertain about climate change risk” groups: older respondents are increasingly less likely to be in the former than those in their 50s, but more likely to be in the latter. For instance, after adjustment for other variables, those aged 80 and older were 13 pp less likely to be classified as “Highly engaged with climate change risk” but 10 pp more likely to be in the “Risk-aware but fatalistic” group. Women were more likely than men to be “Engaged with climate change risk” (3.8 pp) but less likely to be in the “Climate change risk dismissive” (2.3 pp) or “Risk-aware but fatalistic” (3.3 pp) groups. Having children was not associated with any class of ACCR, whereas being unpartnered was associated with a higher likelihood of being classified as “Risk-aware but fatalistic.” There were also clear socioeconomic differences across the groups: for instance, compared to highly educated respondents, those with middle or low educational qualifications were more likely to be classified as “Ambivalent/uncertain about climate change risk” but less likely to be “Highly engaged with climate risk.” Similarly, those getting along financially alright or with difficulties were about 4 pp more likely to be classified as “Ambivalent/uncertain about climate change risk” or “Risk-aware but fatalistic” and around 4 pp less likely to be “(Highly) engaged with climate change risk.” Whereas optimism was not associated with any group, there were mixed differences in SRH and depression across groups based on attitudes toward climate change risk. For instance, those who rated their health as poor or fair were about 3 pp more likely to be “Risk-aware but fatalistic” or “Ambivalent/uncertain about climate change risk” but less likely to be “(Highly) engaged with climate change risk.” Similarly, those with high depressive symptoms were 2.4 pp more likely to be classified as “Risk-aware but fatalistic” but 3.8 pp less likely to be “Ambivalent/uncertain about climate change risk.” Social and civic engagement, except for membership in religious groups, was strongly related to classes of attitudes toward climate change risk. For instance, respondents who are members of a political party, trade union, and environmental groups, who voted in the last general election, and who volunteered in the month before the interview were 12.8, 8.9, and 7 pp, respectively, more likely to be classified as “Highly engaged with climate change risk” compared to those not engaged with any of those activities. These respondents are also about 5–6 pp less likely to be “Ambivalent/uncertain about climate change risk.” Similar results are also found for having environmentally friendly household items. Finally, the results suggest no significant associations between being interviewed in the summer and the five classes of ACCR.

**Table 4:** Average marginal effects for a multinomial logistic regression model for climate change risk classes.

Characteristics	Class 5	Class 1	Class 4	Class 2	Class 3
	Highly engaged with climate change risk	Engaged with climate change risk	Risk-aware but fatalistic	Ambivalent/uncertain about climate change risk	Climate change risk dismissive
**Age (Ref: 50–59)**					
60–69	−0.032 (0.015)*	0.002 (0.017)	0.015 (0.009)	−0.001 (0.014)	0.013 (0.008)
70–79	−0.066 (0.016)***	−0.000 (0.017)	0.057 (0.010)***	−0.006 (0.015)	−0.002 (0.008)
80 and older	−0.130 (0.019)***	0.012 (0.022)	0.100 (0.015)***	0.021 (0.019)	−0.004 (0.016)
**Female**	0.003 (0.011)	0.038 (0.012)**	−0.033 (0.008)***	0.014 (0.010)	−0.023 (0.006)***
**Unpartnered**	0.001 (0.012)	−0.020 (0.013)	0.022 (0.008)**	0.003 (0.011)	−0.007 (0.006)
**No children**	0.027 (0.014)	−0.004 (0.015)	0.008 (0.011)	−0.025 (0.013)	−0.005 (0.007)
**Education (Ref: High)**					
Middle	−0.186 (0.014)***	0.048 (0.014)***	0.025 (0.008)**	0.110 (0.011)***	0.004 (0.006)
Low	−0.259 (0.017)***	0.003 (0.018)	0.078 (0.012)***	0.157 (0.016)***	0.022 (0.010)*
**Financially, gets along (Ref: Very well)**					
Quite well	−0.020 (0.013)	−0.003 (0.014)	0.002 (0.008)	0.040 (0.012)**	−0.018 (0.006)**
Alright or with difficulties	−0.046 (0.015)**	−0.039 (0.016)*	0.039 (0.011)***	0.043 (0.014)**	0.002 (0.014)
**Poor or fair self-rated health**	−0.037 (0.014)**	−0.032 (0.014)*	0.031 (0.009)**	0.027 (0.012)*	0.010 (0.007)
**Has ≥4 CES-D depressive symptoms**	−0.005 (0.018)	0.028 (0.019)	0.024 (0.012)*	−0.038 (0.014)**	−0.008 (0.008)
**Not optimistic**	0.001 (0.014)	−0.014 (0.014)	−0.011 (0.009)	0.016 (0.012)	0.008 (0.007)
**Member of a political party, a trade union, or an environmental group**	0.128 (0.017)***	−0.066 (0.017)***	−0.003 (0.013)	−0.062 (0.015)***	0.004 (0.009)
**Voted in the last general election**	0.089 (0.015)***	0.020 (0.016)	−0.049 (0.011)***	−0.058 (0.014)***	−0.002 (0.008)
**Member of a church or other religious group**	0.013 (0.015)	0.022 (0.017)	−0.017 (0.010)	−0.020 (0.014)	0.002 (0.009)
**Voluntary work in the previous month**	0.070 (0.015)***	0.009 (0.016)	−0.010 (0.011)	−0.054 (0.014)***	−0.017 (0.007)*
**Has environmentally friendly household items**	0.071 (0.016)***	−0.008 (0.016)	−0.020 (0.011)	−0.036 (0.014)*	−0.006 (0.008)
**Interviewed in the Summer**	0.005 (0.011)	−0.013 (0.013)	−0.007 (0.008)	0.018 (0.011)	−0.001 (0.006)

*Note. N *= 6,572. Standard errors in parentheses. ELSA, Wave 11 (2023/2024).

* *p* < .05.

** *p* < .01.

*** *p* < .001.

## Discussion

This study extends previous research by focusing specifically on older adults, a group rarely examined in latent profile analyses of climate change attitudes, and by employing newly collected ELSA data with items tailored to capture perceived risk, salience, and concern. Unlike prior studies (e.g., Liu et al., 2022), which primarily examined general population samples, our focus allows insights into the heterogeneity of attitudes specifically among older adults. In this study, we segmented older people in England based on their appraisal of several statements about climate change risks. Using LCA, we identified five distinct profiles of attitudes toward climate change: “Highly engaged with climate change risk” (30.3%), “Engaged with climate change risk” (31.3%), “Risk-aware but fatalistic” (11.1%), “Ambivalent/uncertain about climate change risk” (21.5%), and “Climate change risk dismissive” (5.8%). Overall, although to varying degrees, the majority of older people in England were classified as engaged with climate change risk, with only 1 in 17 people aged 50 and older identified as dismissive of the risks linked to climate change. This suggests that, contrary to what has been previously reported, even among older individuals, there is a high level of knowledge and concern about climate change, consistent with other recent national studies (Office of National Statistics, 2024). Our results show that the narrative that older people are generally unaware or unconcerned about climate change and its risks is a simplification that masks a significant range of views within this age group.

It is worth noting that our classification aligns somewhat with the population segmentation of people’s climate change beliefs, concerns, and attitudes found in other studies, even if these were not necessarily focused on older people (Crawley et al., 2020; Kácha et al., 2022; Liu et al., 2022; Milfont et al., 2024; Rhead et al., 2018). For instance, based on nonrepresentative data from a 2018 online survey conducted among British citizens aged 18 and older who resided in the United Kingdom, Crawley et al. (2020) classified respondents into five groups: Highly Engaged (17%), Moderately Engaged (29%), Action-wary (33%), Uncertain (15%), and Deniers (6%). Using data from the UK Household Longitudinal Study in both 2012/2014 and 2018/2020 of people aged 16 and older, Liu et al. (2022) identified three distinct attitude clusters, with the latest data classifying 44% as Concerned, 41% as Paradoxical, and 15% as Skeptical. Across Europe, Kácha et al. (2022) identified four groups by climate change attitudes and beliefs using data collected among people aged 15 and older in 2016/2017 from 23 countries of the European Social Survey: Engaged (18%), Pessimistic (18%), Indifferent (42%), and Doubtful (21%). Using the 2018 New Zealand Attitudes and Values Study, Milfont et al. (2024) classified respondents as Climate Believer Concerned (60.4%), Moderate Climate Believer Concerned (19%), Somewhat Climate Believer Concerned (8.7%), Undecided/Neutral (8.2%), and Climate Skeptics (3.7%). Although the number of groups and percentage of people in each profile change by study, these might be due to differences in the questions used (i.e., number of items, wording of questions, or answer options), the timing of data collection (with some studies dating back to 2009), the samples used (including their representativeness and age range), and the techniques used to identify segmentation profiles (mainly LCA and *k*-means cluster analysis).

Despite these differences, most studies, including ours, suggest that most people belong to a group that believes climate change is happening and has not been exaggerated, viewing it as severe and salient enough to warrant action. This is strongly indicative of potential support for urgent climate change mitigation efforts and readiness to engage with this issue. Only a small percentage of older people dismiss climate change and minimize its adverse effects; this group is therefore the least likely to support actions to mitigate them. However, more than one in five respondents in our study were classified as “ambivalent/uncertain”; this group, which consistently chose the midpoint of the agreement/disagreement scale, may include a mix of genuinely neutral individuals, uncertain respondents, or those disengaged from the topic who have not given climate change and its associated risk much thought. Additionally, our study found about 11% of respondents, mainly people in their 70s and aged 80 and older, who are aware of the adverse consequences of climate change but believe that it is too late to do anything about it and that worrying about these effects is pointless, as they are too distant in the future. This group, here defined as “Risk-aware but fatalistic,” is likely to feel they lack agency (or sufficient time) to make any difference, either through individual actions or by endorsing public policies. It is often suggested that emotional engagement, especially worry, is a key driver of involvement in personal and collective efforts to undertake the transformational changes necessary to mitigate and adapt to climate change (Bouman et al., 2020). Policymakers and activists interested in shifting those individuals from an ambivalent/uncertain stance to emotional engagement with climate change might have to focus on making this issue more relatable, highlighting how it could personally affect them and their communities (both directly and indirectly) in the short term (Bouman et al., 2020; Huang & Guo, 2024; Morris et al., 2020). However, it is also essential that climate change is framed as a *challenging yet solvable* problem, avoiding overly optimistic messaging, to ensure that even fatalistic older people engage in behaviors or support policies to address climate change (Mayer & Smith, 2019).

In this study, we also investigated factors associated with profiles of ACCR. We found evidence suggesting that people aged 70 and older are more likely to be classified as “risk-aware but fatalistic,” while those in their 50s are more likely to be “highly engaged with climate change risk.” However, age groups were not associated with the other three ACCR profiles, highlighting that the relationship between age and climate change perception is more complex than previously suggested (Crawley et al., 2020; Rhead et al., 2018; Shwom et al., 2010). This could be driven by the fact that beliefs, worry, and the salience of climate change risks are affected, in complex ways, by age, cohort, and the length and period of exposure to the issue (Johnson & Schwadel, 2019). For example, the greater prevalence of the “risk-aware but fatalistic” group among older respondents may reflect both age effects, such as perceptions of limited personal agency, and cohort effects, given that climate change became a salient issue relatively late in their lives and life stages. Moreover, as people age, socializing during periods of greater climate awareness and heightened environment-related issues on the political and public agenda, one may expect views held by future cohorts of older adults to differ substantially from those most prevalent in today’s older population. Future studies should use longitudinal or repeated cross-cohort data to monitor and account for the complex interplay of age, period, and cohort effects in relation to ACCRs across age groups. In line with recent studies (Liu et al., 2022, 2024; Milfont et al., 2024), we also found that, overall, more affluent older people (i.e., with more financial and educational resources) are more likely to be highly engaged with climate change risk and less likely to be ambivalent/uncertain about climate risk or fatalistic. It has been suggested that having adequate resources increases access to information about climate change and its potential consequences, leading to better awareness of the urgency to act, as well as the financial resources necessary to mitigate their carbon footprints through high-cost, climate-friendly technologies and practices, such as electric vehicles or heat pumps. Furthermore, even in our study, we found that being engaged in activities such as volunteering, voting, or being a member of a political party, trade union, or environmental group is associated with a higher likelihood of being highly engaged with climate risk, and a lower likelihood of being “ambivalent/uncertain” about this issue. This high level of concern about climate change among socially engaged individuals may be driven by several factors, including a greater awareness and knowledge of societal problems, as well as a heightened concern for the well-being of others (Khatibi et al., 2021). Finally, it is worth mentioning that, in our study, we did not find a clear characterization of older people who were climate-risk dismissive. Overall, it is worth noting that although this study focused on people aged 50 and older, and our classification of ACCR groups differs from that of other studies, we also found some evidence of heterogeneity: younger older adults, those who are better-off, more highly educated, and publicly/socially engaged tend to be more informed and concerned about the saliency of climate change and its risks.

### Strengths and limitations

To our knowledge, this study is the first to investigate distinct profiles of attitudes toward climate change risk among older people, participants of a large nationally representative survey of older people in England (ELSA). The richness and breadth of the ELSA data set enabled us to examine a wide range of sociodemographic, psychosocial, and health-related factors associated with these attitudinal profiles, offering insights into how best to tailor public engagement and educational campaigns for this population. Moreover, our findings are based on newly collected climate change questions, ensuring that the results are both timely and highly relevant to current policy and public discourse. Our contribution, however, should be considered in light of some limitations. Questions about ACCR (as well as on social engagement) were included in the self-completion questionnaire. Supplementary Table 2 shows that respondents who returned this questionnaire were, on average, older, financially better off, healthier, and more engaged than the Wave 11 sample, and this could potentially influence the prevalence of ACCR classes. Moreover, the data used were designed in the context of a broad national survey, with questions about climate change limited to one module of the study and not covering other vital aspects relevant for ACCR, such as the degree of involvement in this issue, energy use behaviors, preferred societal responses to this crisis, or why they hold specific views about climate change. Also, we acknowledge that having environmentally friendly household items may be influenced by one’s levels of belief, worry, and seriousness of climate change (rather than reflecting a tangible commitment to environmental responsibility). Similarly, although climate knowledge is one of the strongest predictors of support for both behaviors and policies that help mitigate against climate change, ELSA did not ask questions about the level of understanding of the causes and consequences of climate change. Furthermore, ELSA did not collect information about engagement with social media and news, particularly about climate change. Additionally, although we controlled for respondents’ voting in the most recent election, political affiliation was not collected in ELSA. Membership in political parties or religious groups does not necessarily capture the actual engagement of older people with these organizations. Also, although individual views of climate change risk might reflect personal experiences of heatwaves, flooding, wildfires, or other climate-related events, no such information was available in ELSA. It is also likely that their responses may be influenced by social desirability bias, although the use of self-completion questionnaires likely lowered the likelihood that participants provided normatively desirable responses. Finally, because we used a two-step approach (LCA followed by regression on assigned class membership), parameter estimates may be attenuated due to classification error.

## Conclusion

In conclusion, our study provides an overview of the heterogeneity in attitudes toward climate change risk among older people in England. Based on a nationally representative study, our findings indicate that most older people are concerned about climate change, although there is notable heterogeneity, with just over one quarter of them remaining “Ambivalent/uncertain” (21.5%) or “Dismissive” (5.8%) about this issue. Policymakers and climate activists or organizations should tailor their campaigns to these groups, ideally avoiding a “one size fits all” approach in their communication and education campaigns, if they want to further awareness of this issue and increase support for policies aimed at mitigating climate change.

## Supplementary Material

gbag029_Supplementary_Data

## Data Availability

The data underlying this article are available in the UK Data Archive at https://ukdataservice.ac.uk/ and can be accessed with SN 5050.
