# Supplementary material for: Attitudes toward Climate Change Risk among Older People: New Evidence from the English Longitudinal Study of Ageing
Source: J Gerontol B Psychol Sci Soc Sci. Author manuscript; Available in PMC 2026 Mar 25. (PMC13009027; doi:10.1093/geronb/gbag029)
Supplement: Supplementary file 1 [file NIHMS2155857-supplement-Supplementary_File.docx]

**Supplementary Table 1. Comparison of goodness-of-fit criteria for latent class analysis of different sizes**

| **N classes** | **AIC** | **BIC** | **cBIC** | **Entropy** | **Class membership (%)** | **Average Latent Class Probability (%)** |
| --- | --- | --- | --- | --- | --- | --- |
| 1 | 113204.3 | 113368.1 | 113291.8 | 1 |  |  |
| 2 | 102726.8 | 103061.2 | 102905.5 | 0.829 | 45.6; 54.4 | 94.6; 95.3 |
| 3 | 100102.2 | 100607.2 | 100372.1 | 0.786 | 36.1; 21.4; 42.5 | 94.1; 89.6; 87.7 |
| 4 | 98485.7 | 99161.3 | 98846.7 | 0.807 | 20.6; 6.8; 31.2; 41.4 | 87.3; 89.2; 93.4; 88.2 |
| **5** | **97456.8** | **98303.1** | **97909.1** | **0.807** | **31.3; 21.5; 5.8; 11.1; 30.3** | **85.3; 87.4; 88.9; 81.3; 94.2** |
| 6 | 96981.3 | 97998.2 | 97524.6 | 0.791 | 16.0, 3.3, 32.1, 7.6, 30.1, 10.9 | 83.7; 90.1; 84.5; 82.8. 90.4; 80.4 |
| 7 | 96509.3 | 97696.8 | 97143.9 | 0.782 | 8.9; 6.2; 16.6; 3.2; 27.2; 9.3; 28.4 | 79.2; 84.8; 77.6; 91.2; 82.5; 81.0; 90.4 |

Source: ELSA waves 11 (N=6723). Notes: AIC=Akaike Information Criterion; BIC=Bayesian Information Criterion; c-BIC=sample size corrected BIC

**Supplementary Table 2. Descriptive statistics comparing characteristics of full ELSA respondents and analytical sample (2023/2024)** 

|  | **Percentage** % **(N)** |  |  |
| --- | --- | --- | --- |
|  | **Analytical Sample** | **All ELSA** | **P value** |
| Age:  50-59 | 21.8 (1,431) | 23.3 (1,805) | <0.001 |
| 60-69 | 32.9 (2,163) | 32.7 (2,504) |  |
| 70-79 | 31.6 (2,074) | 30.1 (2,331) |  |
| 80 and older | 13.7 (904) | 14.3 (1,107) |  |
| Mean Age (SD) | 68.54 (9.67) | 68.27 (9.88) | <0.001 |
| Female | 56.3 (3,704) | 55.3 (4,284) | <0.001 |
| Partnered | 64.5 (4,242) | 64.8 (5,019) | 0.092 |
| No children | 17.0 (1,117) | 16.7 (1,291) | 0.182 |
| Education: Low | 20.3 (1,335) | 20.8 (1,610) | 0.449 |
| Middle | 49.1 (3,228) | 48.8 (3,769) |  |
| High | 30.6 (2,009) | 30.4 (2,349) |  |
| Financially, Gets along: Very Well | 46.2 (3,036) | 44.5 (3,433) | <0.001 |
| Quite Well | 30.7 (2,018) | 30.7 (2,372) |  |
| Alright or with Difficulties | 23.1 (1,518) | 24.8 (1,919) |  |
| ≥ Good Self-Rated Health | 72.9 (4,794) | 71.7 (5,252) | <0.001 |
| ≤ 3 CES-D Depressive Symptoms | 86.1 (5,664) | 85.5 (6,189) | <0.001 |
| Voluntary Work in the previous month | 17.0 (1,117) | 15.6 (1,206) | <0.001 |
| Has Environmentally Friendly Household Items | 14.8 (972) | 14.6 (1,131) | 0.810 |
| Interviewed in Summer | 28.8 (1,890) | 29.2 (2,264) | 0.032 |
|  |  |  |  |
| N Respondents | 6,572 | 7,747 |  |

Source: ELSA, W11 (2023-2024).
